# Complete Genome Sequence of a Newcastle Disease Virus Strain Belonging to a Recently Identified Genotype

**DOI:** 10.1128/genomeA.00100-12

**Published:** 2013-01-24

**Authors:** François-Xavier Briand, Aurélie Henry, Paul Brown, Pascale Massin, Véronique Jestin

**Affiliations:** Anses, Ploufragan/Plouzané Laboratory, Ploufragan, France

## Abstract

We report the first complete genome sequence of a strain that presents some pathogenicity and that belongs to a recently characterized genotype of avian paramyxovirus type 1 (APMV-1). This virus, isolated from the common teal, presents the most divergent genome within class I of APMV-1.

## GENOME ANNOUNCEMENT

Newcastle disease is a serious infectious disease in poultry, and outbreaks must be reported to the World Organisation for Animal Health (OIE) (1). This disease is caused by avian paramyxovirus type 1 (APMV-1), which belongs to the family *Paramyxoviridae*, the subfamily *Paramyxovirinae*, and the genus *Avulavirus* (2). This virus has a negative single-stranded RNA genome of 15,186 to 15,198 nucleotides encoding 6 major proteins (3). APMV-1 is divided into 2 classes based on genetic analysis. Class II strains have been recovered from wild or domestic birds, include virulent and avirulent isolates, and are divided into different genotypes (3–6). Class I strains have been isolated mainly from wild birds and are avirulent (except for one isolate; see reference 7). Class I strains are divided into nine genotypes (8); however, in a recent study, these have been condensed into a single genotype (6). During active surveillance of avian influenza in 2009 in France, APMV-1 (Teal/France/100011/2010) was isolated in 9-day-old embryonated specific-pathogen-free chicken eggs from cloacal swabs collected from apparently healthy common teal (*Anas crecca*). Viral RNA was extracted from allantoic fluid and was reverse transcribed to yield cDNA using SuperScript II (Invitrogen). The complete genome was obtained by overlapping PCRs using Platinum *Taq* high-fidelity DNA polymerase (Invitrogen). PCR products were sequenced with an Applied Biosystems 3130 Sanger-based genetic analyzer. Contigs containing high-quality trace files were assembled using Vector NTI software (Invitrogen). The genome extremities were acquired using a novel rapid amplification of cDNA ends (RACE) strategy (P. Brown et al., submitted for publication). The genome sequence was found to be 15,198 nucleotides in length, respecting the rule of six of paramyxovirus, and its organization of nucleoprotein/phosphoprotein/matrix protein/fusion protein/hemagglutinin-neuraminidase/large protein (NP/P/M/F/HN/L) was in agreement with all APMV-1 strains. The NP, M, F, and L amino acid sequences deduced from the genome sequence were in agreement with the expected sizes of class II and class I APMV-1 proteins, with 489, 364, 553, and 2,204 amino acids (aa), respectively (4). The P was 399 aa long, a trait which is specific to class I viruses, in contrast to the class II viruses, which have Ps 395 aa long (4). The HN protein was 616 aa long, which is the longest for APMV-1. Phylogenetic analysis of the complete genome sequence using the neighbor-joining method confirmed that the virus Teal/France/100011/2010 was closely related to the class I viruses. Nevertheless, the distance of the genome sequence from those of other class I viruses was greater than any of the distances among other class I virus genomes. The nearest genome was that of Goose/Alaska/415/1991, with 83.9% nucleotide identity. The nearest class II virus was Mallard/United States(MN)/MN00-39/2000, with 73.3% nucleotide identity. The virus studied here belongs to the recently identified genotype 10 of class I (9) and could belong to a new genotype, genotype 2, of class I, according to the new classification (6). The cleavage motif site, which corresponds to the major evidence for APMV-1 virulence, was deduced from the F sequence and was ^111^ERQERL^117^, corresponding to that of the avirulent virus; the average values obtained (0.75) using the intracerebral pathogenicity index were slightly superior to the threshold between the avirulent and virulent viruses, according to the OIE (1). This report will facilitate investigations into the molecular pathogenesis of APMV-1 infection.

### Nucleotide sequence accession number.

The genome sequence of the Newcastle disease virus (NDV) strain has been deposited in GenBank (accession no. JQ013039).
